# Division of childcare policy actors under health-oriented goals: thematic analysis of China’s policy texts from the social constructionist perspective

**DOI:** 10.3389/fpubh.2024.1454537

**Published:** 2024-12-12

**Authors:** Linan Li, Junyu Li

**Affiliations:** ^1^Research Center of Chinese Village Culture, Central South University, Changsha, China; ^2^Institute of Guangdong, Hong Kong and Macao Development Studies, Sun Yat-sen University, Guangzhou, China

**Keywords:** health-oriented goal, childcare policies, policy actor, social construction, China

## Abstract

**Background:**

Ensuring child health, as a key objective of global childcare policies, requires coordinated efforts between the government, social organizations and communities, institutions, and families. Despite China’s progress in comprehensive childcare policy development, rapid economic growth, and urbanization, challenges persist, such as urban–rural disparities and unequal resource distribution, highlighting the need for effective collaboration between policy actors.

**Methods:**

To collect textual data, this study searched for prefectural-level childcare policy texts issued since 2019 on government websites and legal databases, ultimately identifying 224 documents for analysis. This study reviewed the literature on the impact of childcare policies on child health and identified the enhancement of childcare quality as a current research focus. This study then conducted a content analysis using Nvivo12 Plus software and coded and analyzed the childcare policy content. Finally, it applied social construction theory to interpret the policy documents.

**Results:**

Childcare policies were centered around child health and formed a responsibility and accountability framework between the government, social organizations and communities, institutions, and families, whose action shares accounted for 38.9, 22.89, 29.05, and 9.16%, respectively. The development of childcare institutions was a key aspect of the defamilialization trend. Compared to other policy actors, institutions played a larger role in child health policy aspects such as safety management (12.97%), health and hygiene (8.56%), and scientific parenting (10.93%).

**Conclusion:**

Within China’s health-oriented framework, the refamilialization and defamilialization processes coexist in terms of childcare policies, and limited community-based childcare resources extend beyond the family. The participation of diverse policy actors in China’s childcare system is expected to persist, underscoring the increased need to enhance the policy actors’ negotiation skills and bolster community-based childcare services in the future.

## 1 Introduction

Child health is a global priority that impacts physical and psychological wellbeing and shapes social welfare and development potential in the future. Consequently, promoting child health is a key objective in the implementation of childcare policies worldwide (1). China, as one of the most populous nations, has introduced specialized childcare policies that emphasize child health and advocate for the involvement of diverse stakeholders. This approach benefits countless Chinese families and children and provides an informative model for other developing nations, meriting closer scholarly investigation.

The United Nations (UN) Convention on the Rights of the Child (CRC) forms a significant foundation of global childcare policy development. Adopted by the UN General Assembly on November 20, 1989, the CRC comprises a comprehensive framework that safeguards children’s rights, health, and wellbeing. It also prioritizes the advancement of children’s physical, psychological, and emotional health through a targeted care policy.^1^ Ratified by 196 countries, the CRC has spurred a worldwide commitment to prioritize children’s needs and rights within national policy frameworks,^2^ and evidence indicates growing global endorsement of childcare policies (2). Globally, childcare policies can be divided into three models based on the policy actors involved. The first type, “optional familialism,” involves government support and resources. It grants families the autonomy to choose between State assistance and private caregiving options, so as to promote diversity and flexibility in its implementation. France, for example, supports families by providing public childcare services, particularly for children aged 0–2 years, when parents still play a considerable caregiving role (3). In Nordic countries such as Denmark, Finland, Sweden, and Norway, early childhood education and childcare services are primarily State funded and are often free or subsidized, ensuring universal access to high-quality care (4–6). The second type, “implicit familialism,” is characterized by minimal governmental support for family caregiving and a lack of policy to ease the caregiving burden. This approach subtly reinforces the family as the primary welfare provider, leaving them with substantial caregiving responsibilities. In response to the inconsistent quality of childcare services and the demand for improved standards, the United States (US) introduced the Quality Rating and Improvement System to assess and improve the quality of early childcare services to support children’s cognitive and emotional development, with implementation across the US by 2017 (7). Meanwhile, East Asian nations are increasingly viewing childcare policies as essential for addressing declining birth rates. Japan, for example, has championed stakeholder engagement through the Comprehensive Support System for Children and Childrearing to foster a cohesive and high-quality early childhood education and childcare environment (8). Meanwhile, South Korea’s childcare policies are increasingly regarding caregiving as a social responsibility and shifting toward family support systems, including formal support for multicultural families (9). The third type, “explicit familialism,” involves deliberate government actions that empower families in their caregiving roles, albeit with limited alternative institutional support. Germany, for example, has introduced dual-earner family policies that encourage parents to balance work and caregiving responsibilities through public services, economic assistance, and flexible work policies, so as to support family caregiving for young children (10). In sum, the predominant trends in global childcare policies emphasize early childhood education and childcare, promote gender equality, and highlight the development of public service-led support systems. These initiatives emphasize the safeguarding of child health a crucial goal in the global implementation of childcare policies. However, achieving these health objectives requires the involvement of family units alongside effective collaboration between and support from various social actors (11, 12). Therefore, examining the roles of different policy actors within the childcare policy framework can enhance the understanding of and optimize the system’s capacity to protect child health.

Under the CRC’s influence, China’s childcare policies have evolved along a systematic and refined developmental path and have been continuously updated and improved over time. Based on the CRC, China issued the 1992 *Outline of the Development Plan for Chinese Children in the 1990s* based on national conditions; this was the first national action plan that focused on children and promoted their development. Subsequently, the Chinese government has formulated and implemented the *China Children’s Development Plan* (CCDP) every decade, with four editions published to date. Analyzing the release and execution of the CCDP provides the following valuable insights into the impact of China’s childcare policies on child health over the past 30 years. First, in terms of its overall effectiveness, infant mortality and under-five mortality rates significantly decreased from 51 and 61% in the 1990s to 5.4 and 7.5% in 2020. By 2010, the national immunization program’s vaccination rate had exceeded 90%.^3^ Second, in its formulation of childcare policies, the Chinese government and relevant agencies have consistently adhered to the “child first” principle, prioritizing “children and health” as the central goal. Third, the focus of China’s childcare policies has evolved over time, shifting from birth rates and disease prevention to the development of comprehensive child health service systems. These changes reflect the significant progress taken to reduce infant mortality and expand vaccination coverage. Driven by rapid economic growth, accelerated urbanization, and declining birth rates, the Chinese government has recently introduced and revised more holistic childcare policies that address healthcare, childcare services, nutrition, and safety. On May 9, 2019, the General Office of the State Council of China released the *Guiding Opinions on Promoting the Development of Care Services for Infants and Young Children Under 3 Years Old* (GOPD), which outlines the fundamental principles, goals, and tasks for advancing childcare services.^4^ This is the first instance in which the Chinese government has established a dedicated policy for infant and childcare rather than addressing it within the broader child development scope. Additionally, the GOPD is the first policy to clarify the specific childcare responsibilities of government departments and emphasize the shared caregiving roles between families, the government, institutions, and social organizations and communities. Under the GOPD’s guidance, local governments have begun to more intensively issue childcare policies, marking a shift in China’s approach toward greater childcare systematization and refinement.

However, challenges persist, including disparities in care quality, non-disease-related health issues, and insufficient childcare resources, which families alone cannot adequately manage. Compared to general hospitals, children’s hospitals are better equipped to offer community parenting support by tackling issues such as nursing, substance abuse, social needs, chronic disease management, and mental health (13). Additionally, overfeeding during the neonatal period has been linked to accelerated weight gain in children aged under 2 years, which increases the risk of childhood obesity (14). Therefore, it is imperative to develop robust public health policies that regulate early childhood nutrition. Moreover, pronounced disparities exist in the policy implementation between urban and rural areas, such as rural children having inadequate access to educational and healthcare resources (15), and infants aged 6–23 months failing to receive the minimum dietary intake from breastfeeding and complementary feeding, leading to increased morbidity with age (16). Further, the quality and safety of certain childcare facilities require enhancement, while childcare services in specific regions remain inadequate (17). While these urgent issues necessitate the involvement of various policy actors, they remain to be effectively addressed, highlighting the need for a reevaluation of the current childcare policy framework. Accordingly, this study explores the following questions: How do different childcare policy actors (i.e., the government, social organizations and communities, institutions, and families) allocate responsibility within the health-oriented framework? How have the roles of the family and non-family sectors evolved within China’s childcare policy system?

The current research on the division of responsibilities among childcare policy actors in childcare policies has predominantly viewed childcare as the responsibility of individual actors, emphasized gender roles within families, and highlighted women as primary caregivers (18–20). Within the family, women share childcare responsibilities through two avenues. The first involves men’s participation in childcare. Influenced by traditional gender roles and sociocultural norms, men often assume the provider role, which leads to their childcare contributions being marginalized and less visible (21). Social policies and institutional frameworks reinforce this dynamic, resulting in a rigid pathway to paternal practices (22). As more women enter the workforce and contribute to household incomes, paternal childcare involvement has gradually increased (23). However, men continue to face significant barriers in terms of engagement, including the need to overcome entrenched social perceptions (24). To address these issues, there is a need for supportive social policies that promote paternal involvement (25). The second avenue comprises the intergenerational care model. This model is prevalent among Chinese middle-class families, where grandparents collaborate on childcare to alleviate and redistribute family child-rearing pressure (26). However, this model introduces social pressure; for example, older caregivers may experience stress that can negatively impact child health (27, 28). Meanwhile, some studies have acknowledged that families cannot independently shoulder the childcare burden. When social policies integrate the protection of women’s employment with childcare improvements, policy interventions can effectively alleviate women’s work–family conflict (29) by assuming some responsibilities; however, the relationship between the government and families remains in flux (30). Moreover, childcare development initiatives are currently fragmented across various government sectors, including health, nutrition, education, childcare, and social security. To ensure that interventions are effective, well-informed, and sustainable, it is essential to adopt a child health-centered approach that fosters multisectoral collaboration (31). Additionally, the successful implementation of childcare policies necessitates the involvement of diverse policy actors. The extant research has highlighted the roles of market entities and civil society as intermediary forces in terms of childcare. However, for-profit childcare institutions encounter challenges related to market failure and information asymmetry (32), while community childcare institutions require systematic resource mobilization (33). Accordingly, notable research gaps exist: first, the roles of other childcare policy actors have not been thoroughly considered, including institutions and social organizations. Moreover, systematic interventions by multiple actors warrant further investigation. Second, the research has failed to examine the division of labor and the significance of policy actors in terms of child health objectives.

Accordingly, this study analyzes China’s childcare policy texts to explore the allocation of childcare responsibilities between different policy actors (i.e., governments, social organizations, institutions, and families) in relation to various health-related tasks. Specifically, this study analyzes 224 childcare policy documents issued by Chinese prefectural governments since 2019 and treats them as a distinct policy system. These documents encompass multiple regions and reflect diverse local conditions, so as to capture the complexities and variations inherent in the policy implementation process. These also provide a nuanced and comprehensive representation of the division of responsibilities among the different policy actors within China’s childcare sector. Utilizing a qualitative content analysis, this study then employs NVivo software to code the childcare policy texts. Guided by social construction theory, this study establishes a two-dimensional analytical framework that positions policy actors as the X-dimension and child health as the Y-dimension. This framework facilitates the examination of different policy actors’ specific actions concerning child health objectives through the coding, categorization, and quantification of their actions within the childcare policy texts, enabling the systematic analysis of different policy actors’ hierarchical roles in meeting child health objectives.

In so doing, this study contributes to the literature in three ways. First, it identifies the coexistence between refamilialization and defamilialization trends in China’s current childcare policies. Consequently, this study argues that the government must better coordinate the responsibilities of family and non-family actors by increasing financial subsidies and providing training support for childcare institutions; doing so will prevent families from shouldering an excessive share of childcare duties. Second, this study highlights the insufficient role that communities play in child health objectives related to safety management, health and hygiene, and scientific parenting. Consequently, this study advocates for the establishment of inclusive, community-based childcare centers in urban and rural areas. These centers must offer accessible and supplemental services that are closer to family homes, so as to address disparities in healthcare quality, standards, and childcare resources. Finally, this study emphasizes the importance of communication between different policy actors and proposes the creation of a platform for them to engage in collaborative dialog. Doing so will allow the government to better understand families’ childcare needs and monitor the quality of services provided by social organizations and institutions.

## 2 Methods

### 2.1 Theoretical perspective

This study analyzed childcare policy texts using social construction theory, which refers to the prominent interactive construction and configuration features of the relationship or association between individuals and society (34). This study’s application of social constructionism focused on two aspects. First, the diverse multisubject participation in the construction of child health. During this process, different subjects consciously or unconsciously play different roles in shaping, configuring, and influencing the original meaning and nature of others’ actions (35). In China, childcare is diverse and mainly undertaken by families (36). Accordingly, the government has begun to formulate corresponding policies to meet families’ childcare needs. With the encouragement of government policies, institutions have gradually joined in the provision of childcare, while social organizations and communities have formed constraints and supplementary childcare (37).

The second aspect emphasizes the practical social construction process of child health, which involves generating, practicing, and reflecting on multiple meanings, as follows (38). First, diverse subjects participating in childcare form action meanings and interact with each other during the interaction and construction processes. The transformation from subjective to external action meanings means that relevant policies are adjusted because the significance of child health is valued. Further, iterative adjustment and reflective monitoring (e.g., the examination, verification, and identification of problems in childcare policies by institutions, social organizations and communities, and families) enables the government to grasp new information and adjust, revise, and innovate policies accordingly (39).

### 2.2 Document selection

**Figure 1:**
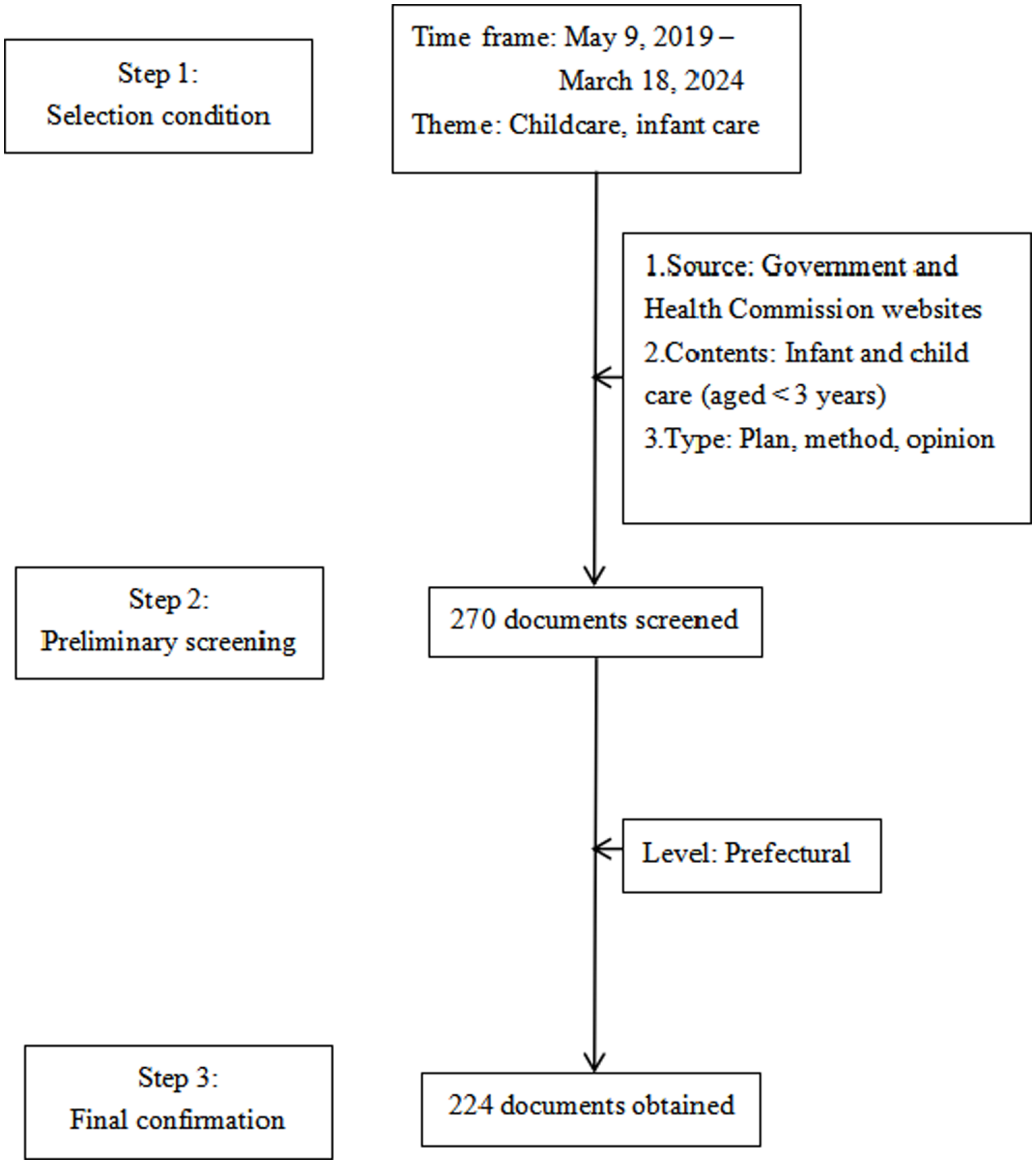
Childcare policy text screening process.

This study employed a systematic approach to the text selection via selection criteria, a preliminary screening, and the establishment of coded texts (Figure 1), as follows.

Step 1: this study searched for policy texts from May 9, 2019–March 18, 2024. On May 9, 2019, the General Office of the State Council of China issued the GOPD and directed local governments to develop feasible policy measures within this framework. This event marked China’s initiation of the development of childcare policy as an independent system. Therefore, this study designated 2019 as the baseline year for the independent launch of childcare policies and concentrated on the initiatives introduced in various cities and provinces after May 9, 2019. The end date aligned with the most recent urban childcare policy implementation plan (the *Inclusive Childcare Service Development Demonstration Project*) released by the People’s Government of Changzhi City, Shanxi Province, on March 18, 2024.^5^

Step 2: this study used the “childcare” and “infant and toddler care” search terms to conduct a preliminary screening, with a focus on the development of childcare services for children aged under 3 years. The research team established three selection criteria: (1) policies issued by the People’s Government and Health and Hygiene Committees; (2) policies containing relevant provisions for developing care services for the target age group; and (3) documents that included plans, measures, and opinions but excluded responses, directives, and policy interpretations. To ensure the comprehensiveness and authority of the policy text sources, this study screened 270 documents using the aforementioned search terms across the official websites of national, provincial, and prefectural governments; health departments; and legal databases, such as *Peking University Law Treasure* and *Peking University Law Meaning*. This collection comprised 16 national-, 30 provincial-, and 224 prefectural-level documents (Table 1). All policy documents were in Chinese, so this study translated them into English for the subsequent analyses.

**Table 1:** Hierarchical distribution of preliminary screening policy texts.

	2019	2020	2021	2022	2023	2024
National level	3	2	2	5	3	1
Provincial level	11	18	0	0	1	0
Prefectural level	9	147	37	22	6	3

Step 3: this study focused on prefectural-level documents for the coding analysis. Since the number of national-and provincial-level texts was limited, they served as contextual supplementary material. The rationale for selecting prefectural-level texts as the coding sample was twofold. First, in terms of quantity, prefectural-level texts provide a substantial dataset for childcare policy compared with those from the other levels. Second, prefectural-level texts reflect the direction established by national and provincial policies and detail the specific actions undertaken by various policy actors. Consequently, this study identified 224 prefectural-level policy texts as the analysis subject and study database. The descriptive statistics and coding analysis were based on these texts.

To ensure the consistency and generalizability of the analysis, the sample excluded China’s four municipalities that fall directly under the central government (Beijing, Shanghai, Tianjin, and Chongqing). As provincial-level administrative entities, these municipalities have policy development and implementation processes similar to those of provinces and autonomous regions, resulting in significant differences when compared to prefectural-level cities, which could compromise the consistency and comparability between the municipal-and prefectural-level policies. Further, these four municipalities generally have higher levels of economic development and resource allocation, allowing them to more robustly support childcare services. Therefore, the division of childcare policy roles may fail to accurately represent broader national trends. Consequently, this study excluded these four municipalities from the sample and analyzed the 224 prefectural-level texts.

### 2.3 Study design

This study used Nvivo12 Plus software to analyze the 224 texts via open, axial, and selective coding (40). NVivo 12 Plus is suitable for analyzing various unstructured data, including text, video, and audio, as it possesses robust data coding and theoretical model-building capabilities, enabling the efficient and precise retrieval and coding of large datasets and the visualization of results within a systematic and scientific framework.

This study conducted a content analysis to provide an objective, systematic, and quantitative analysis of the policy texts (41). The essence of a content analysis lies in its scientific and rigorous coding of textual content, leading to in-depth qualitative conclusions. It serves as a powerful tool for exploring social phenomena, interpreting meanings, and uncovering both overarching and deep-seated social and cultural structures; therefore, this study deemed it suitable for use. This study then developed a two-dimensional analytical framework based on the content analysis of the policy texts, focusing on the policy actors and child health objectives. First, this study used open coding to analyze the policy actors’ specific actions (the X-dimension). Then, this study used axial coding to determine their actions in relation to child health (the Y-dimension). This two-dimensional approach elucidated the division of responsibilities among different childcare policy actors within the health-oriented framework to highlight the development patterns of China’s childcare policies. This process involved three steps.

Step 1: open coding. This study situated the specific action strategies outlined in policy texts within the hierarchical roles of social policy while further abstracting these strategies to their respective domains, which gradually emphasized the division of responsibility among the different policy actors (Table 2). The open coding process comprised three stages. First, this study coded the policy actions by extracting the child health actions from policy texts and identifying their coding elements, resulting in 28 primary codes. Second, this study focused on coding at the hierarchical level. Policy actors’ hierarchical roles referred to the distribution of responsibilities and functions of actors during policy formulation, implementation, and evaluation (42). By aligning these hierarchies with the actors’ corresponding policy actions, this study established seven categories as secondary codes. Finally, the coding process categorized the respective domains. Broadly speaking, contemporary civil society consists of four sectors: the government, private entities, the public sphere, and institutions (43). The effective functioning of social policies relies on collaboration between these sectors. By mapping the secondary codes of the relevant policy actor domains, four core categories emerged: the government, social organizations and communities, institutions, and families; these constituted the tertiary codes.

**Table 2:** Open coding (X-dimension).

First-level codes	Secondary codes	Tertiary codes
Direct regulation, formulate development plans, formulate industry standards	Organizer	Government (national domain)
Ensure safety, ensure hygiene, oversee and manage, construct information infrastructure	Person in charge	
Provide financial support, develop public services, implement tax incentives, train childcare professionals, provide land support	Resource provider	
Protect employment rights and interests, provide maternity leave support, provide public services	Environmental facilitator (employer)	Social organizations and communities (public sphere)
Affordable child care for the masses, community care, volunteer assistance	Provider of public services (NGO and community)	
Establish a model demonstration, offer diversified services, register in compliance with regulations, formulate staff entry regulations, consolidate safety responsibilities, establish a care brand, integrate child care and kindergarten, manage hygiene and health	Market service provider (kindergartens, daycare centers, caregivers, and suppliers of care products)	Institutions (market sector)
Strengthen maternal and child healthcare, study care-related knowledge	Service recipients (parents and other family members)	Families (private sector)

NGO, nongovernmental organization.

Step 2: axial coding. This study viewed the concept of child health in terms of constructing a child health service system with child health at its core. Based on the open coding results, the following categories linked all concepts: safety management, health and hygiene, scientific parenting, and the social environment (Table 3). This study then related these axial codes to the different policy actors’ actions to verify their authenticity and reliability.

**Table 3:** Axial coding (Y-dimension).

Policy excerpt	Unit (Conceptualization)
Strengthening the Responsibility of Safety Entities. Various child care institutions bear the primary responsibility for the safety and health of infants and young children. The provision of child care services must comply with relevant standards and norms such as the *Trial Measures for the Establishment Standards of Child Care Institutions*, *Trial Measures for the Management Standards of Child Care Institutions*, and *Architectural Design Specifications for Nurseries and Kindergartens*, establishing sound safety protection measures, inspection systems, and emergency response plans for unforeseen events.	Safety management
Strengthening guidance and supervision of health care in child care institutions. Diligently implementing the principle of prevention first and combining health care with education, strengthening planned immunizations to ensure all necessary vaccinations are administered, supervising child care institutions to conduct daily morning checks, hygiene and disinfection, isolation of sick children, prevention and management of infectious diseases, preventing and controlling the incidence of infectious diseases, providing guidance on infant and young child dietary nutrition, conducting regular health check-ups for infants and young children, creating a good living environment for infants and young children, and safeguarding their physical and mental health.	Health and hygiene
Enhancing early development guidance for infants and young children. Organizing activities suitable for the physical and mental development characteristics of infants and young children, providing parents and caregivers of infants and young children with guidance on scientific care and related knowledge through activities such as parent–child activities, home visits, parent classes, and expert consultations. This aims to promote comprehensive development in infants and young children in terms of physical growth, motor skills, language, cognition, emotion, and social interaction, and to enhance the scientific parenting capabilities of families.	Scientific parenting
Enhancing social support. Accelerating the construction and renovation of barrier-free facilities and mother-and-child facilities in public places, opening up green channels for services, and providing convenient conditions for infant and young children’s travel and breastfeeding to create a friendly social environment for child care.	Social environment

Step 3: selective coding. Using social construction theory, this study systematically linked the core category of child health to different policy actors, leading to the identification of two key categories (refamilialization and defamilialization) that formed the theoretical framework of how China’s childcare policy affected child health.

## 3 Results

### 3.1 Descriptive analysis of policy texts

**Figure 2:**
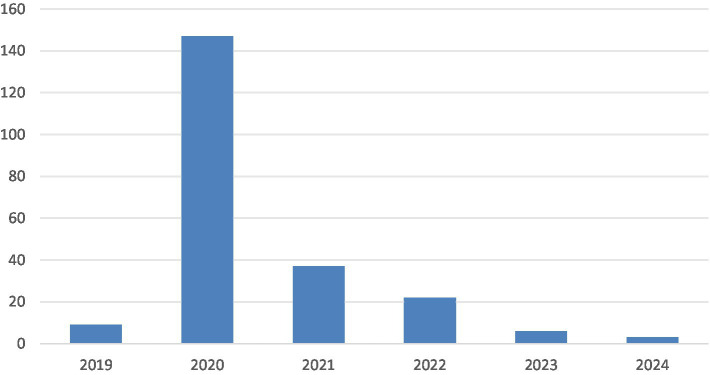
Temporal distribution of prefectural-level policy texts.

**Figure 3:**
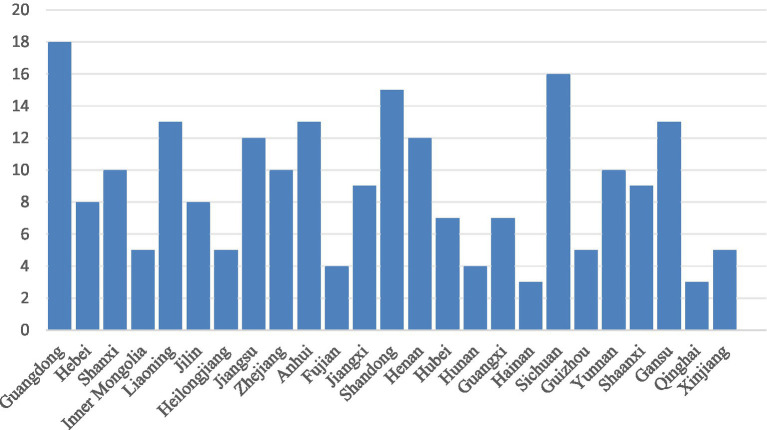
Provincial distribution of prefectural-level policy.

**Figure 4:**
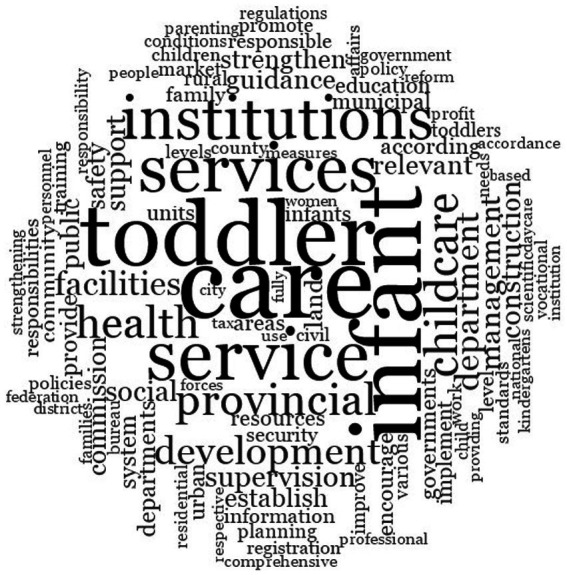
Word cloud of Chinese childcare policy texts.

1. Temporal distribution of policy texts. Figure 2 indicates that the implementation timelines of the 224 prefectural-level childcare policy texts demonstrates an inverted U-shaped trend, with 2020 representing the peak year for policy issuance In China, local governments typically formulate and adjust their policies within a framework established by central government directives and align them with the national agenda’s goals and requirements. Therefore, following the central government’s release of the GOPD in 2019, the local governments promptly responded by introducing childcare policies.
2. Regional distribution of policy texts. Figure 3 shows that the 224 policy texts cover 25 provincial-level administrative regions. This represents a significant proportion of China’s 27 provinces and provincial-level autonomous regions, indicating that most provinces and cities have implemented childcare policies. Regarding the prefectural-level policy text distribution, Guangdong Province (18 cities) has the highest number of implemented childcare policies followed by Sichuan Province (16 cities) and Shandong Province (15 cities).
3. Word cloud analysis of policy texts. This study used NVivo to generate a word cloud visualization of the 224 childcare policy texts. Figure 4 presents the texts’ key characteristics. The word cloud demonstrates over 100 words, and the size of each word reflects its occurrence frequency in the policy texts. Words such as “care,” “infant,” “toddler,” “service,” and “institutions” are the most frequent, confirming that the policy texts primarily focus on childcare services for children aged under 3 years. Words such as “childcare,” “health,” “development,” and “department” also frequently appear, indicating the significant emphasis on child health within China’s current childcare service development landscape for children of this age.

### 3.2 X-dimension: policy actor coding results

This study analyzed the policy texts using axial coding in NVivo with a focus on the different policy actors’ specific actions (the X-dimension). The text analysis and conceptualization revealed actors’ specific actions related to childcare, yielding 3,766 reference points. This study then categorized the different actors’ childcare policy roles. The results (Table 4) show that the government plays the most significant role in childcare (38.9%) and is the responsible entity for and coordinator of policy actions. Its primary responsibilities include enhancing childcare capacity and safeguarding children’s health. This is evident in the development of childcare policies and regulations, the mobilization of resources from various sectors, and the coordination of activities among the diverse childcare policy actors.

**Table 4:** Division of policy actors’ responsibilities.

Policy actors	Policy roles	Code (Percentage)	Sum
Government	Organizer	176 (4.67%)	1,465 (38.90%)
	Person in charge	501 (13.30%)	
	Resource provider	788 (20.92%)	
Social Organizations and Communities	Environmental facilitator	459 (12.19%)	862 (22.89%)
	Provider of public services	403 (10.70%)	
Institutions	Market service provider	1,094 (29.05%)	1,094 (29.05%)
Families	Service recipients	345 (9.16%)	345 (9.16%)

Families that are both demanders and beneficiaries of childcare services play a smaller childcare role (9.16%). Policymakers have recognized the insufficiency of families’ independent caregiving capacity. While efforts have been made to change the traditional caregiving perception (which is predominantly female-centric) through policy advocacy that calls for the participation of all family members, there is a push to engage external policy actors, such as institutions and social organizations, in the provision of childcare.

Finally, the government, social organizations and communities, and institutions constitute considerable childcare service providers. The government offers public resources (20.92%) such as land support and tax incentives; social organizations and communities provide public service support (22.89%) such as flexible parental leave and childcare assistance from public organizations (e.g., nongovernmental organizations); and institutions provide market services for childcare demanders (29.05%) such as nannies or childcare centers. Each policy actor responds to government requirements by supplying appropriate childcare resources (based on their available capacities) to address childcare seekers’ needs; this process creates an institutionalized network of policy actors. Overall, China’s childcare policies prioritize child health and have established a framework for responsibility-sharing and constraints between the government, families, institutions, and social organizations and communities.

### 3.3 Y-dimension: child health coding results

After delineating the policy actors’ specific actions (the X-dimension), this study conducted coding for the Y-dimension (child health). This study identified 1,226 nodes in the two-dimensional coding. Table 5 shows the nodal distribution within each component of the child health dimensions. Overall, safety management comprises 238 nodes (19.41%). In this category, the government focuses on developing and regulating safety management guidelines for childcare facilities (80 nodes), while institutions are responsible for implementing the safety measures (159 nodes). Safety management is fundamental to childcare services and is reflected in four key areas: fire and building safety in childcare centers, enhanced oversight of personal safety for infants and toddlers, strengthened regulation of child food safety, and the enforcement of child safety protection protocols.

**Table 5:** Child health coding results.

Child health	Code	Percentage
Safety management	238	19.41%
Health and hygiene	287	23.41%
Scientific parenting	362	29.53%
Social environment	339	27.65%

Health and hygiene comprises 287 nodes (23.41%) and involves all policy actors’ engagement. Health and hygiene addresses children’s nutritional requirements, disease prevention, and mental wellbeing, and encompasses both public health and children’s physical and emotional health in family care throughout their developmental stages.

Scientific parenting comprises 362 nodes (29.53%). This aspect emphasizes caregivers’ approaches to promoting children’s health. Among the policy actors, childcare practitioners and families comprise the primary contributors (134 and 118 nodes, respectively). The government provides educational support for scientific parenting, such as by training childcare professionals and establishing parenting workshops (109 nodes). The role of social organizations and communities is more limited and primarily involves volunteer efforts to develop and expand teams for infant and toddler care (25 nodes).

The social environment comprises 339 nodes (27.65%). This aims to cultivate a supportive and harmonious atmosphere that fosters both childcare and collaborative efforts toward children’s health. In China’s childcare policies, social environment improvements are evident in terms of the accelerated construction and renovation of accessible facilities, child-friendly amenities in public spaces, and green service pathways that facilitate travel and breastfeeding.

### 3.4 Two-dimensional analysis of policy texts

To further clarify the allocation of responsibilities among the different childcare policy actors, this study utilized a coding matrix query function to analyze the proportions of actions related to child health and the different actors’ policy planning tendencies. Table 6 presents the distribution of policy actors and child health dimensions (X–Y). Under the safety management category, institutions account for the highest distribution (12.97%), and at the national and provincial levels, institutions’ safety arrangements significantly impact child health. In the health and hygiene unit, institutions and families account for 9.71 and 8.56% of the distribution, respectively. Under the scientific parenting category, institutions and families, as direct childcare participants, account for 10.93 and 9.62% of the distribution, respectively. Under the social environment category, social organizations and communities account for 13.62% of the distribution. Overall, the government advocates for a child-friendly environment and encourages other policy actors to participate in policy promotion through the provision of public service facilities. Childcare institutions play a crucial role in ensuring child health and safety management, health and hygiene, and scientific parenting, which are key points for enhancing childcare quality in current and future policies.

**Table 6:** Two-dimensional distribution of childcare policy actors (X-Y).

Policy actor	Safety management		Health and hygiene		Scientific parenting		Social environment	
	Frequency	Percentage (%)	Frequency	Percentage (%)	Frequency	Percentage (%)	Frequency	Percentage (%)
Government	80	6.53	40	3.26	109	8.89	125	10.20
Social Organizations and communities	0	0	23	1.88	25	2.04	167	13.62
Institutions	159	12.97	105	8.56	134	10.93	50	4.08
Families	0	0	119	9.71	118	9.62	0	0

## 4 Discussion

### 4.1 Roles of policy actors

This study used social construction theory to examine the division of childcare roles between different policy actors (i.e., the government, social organizations and communities, institutions, and families) under China’s evolving childcare policy landscape. The prior research (34) contends that roles embody two levels of institutional order: first, through the specific expression of a role that reflects its core identity; second, through a role that encapsulates a comprehensive institutional network related to behavior (34). Therefore, in terms of China’s childcare policies, the government, institutions, families, and social organizations and communities play different roles but are united under an institutional network. As a policy decision-maker, the government is responsible for regulating and managing child health through legislation, policy formulation, and resource allocation. The government’s interest lies in safeguarding the country’s future development, and child health serves as the foundation for achieving this goal. Accordingly, the government invests resources in healthcare, education, poverty alleviation, and other services to ensure the safety and hygiene of childcare environments, bolster scientific knowledge of childcare personnel, eliminate child health risks, and generally ensure child health. Therefore, the government must consider the needs and interests of other policy actors, such as social organizations and communities, families, and institutions, to create a supportive and friendly social environment for childcare.

As policy coordinators, social organizations and communities play a crucial role in child health by providing various services and support. Their interests lie in advocating for vulnerable groups, promoting social equity and justice, and advancing child health and development through various projects and activities, so as to create a supportive social environment for the implementation of childcare policies. Social organizations and communities typically rely on government funding and social donations; therefore, they must collaborate with the government and other institutions to jointly promote the development of child health initiatives.

As policy implementers, childcare institutions are among the key organizations that provide childcare and educational services. They bear the responsibility for child guardianship and education and contribute to child health and wellbeing. Childcare institutions’ interests lie in providing high-quality services to meet parents’ needs while ensuring child health and safety. Therefore, they must collaborate with the government and comply with relevant laws and policies to ensure the quality of services and protection of children’s rights.

As policy beneficiaries, families are fundamental for ensuring children’s growth and development, and they directly influence and bear responsibility for their children’s health. Families’ interests lie in nurturing healthy and happy future generations and ensuring that they receive adequate care and education. Therefore, families require support from the government and institutions, particularly in terms of scientific parenting knowledge, to contribute to healthy child development. This support will enable families to better fulfill their responsibilities in terms of nurturing and educating their children.

In sum, the government, institutions, families, and social organizations and communities play crucial roles in fostering interdependence and cooperation to advance child health and wellbeing. This involves implementing multi-departmental interventions centered on child health and calling for social organizations and communities to provide childcare services (31, 32). Each actor undertakes specific actions at various policy development, implementation, and adjustment stages that are guided by their interests, roles, and influences (44). This division of roles reveal distinct trends in policy development (45). By focusing on child health, policy objectives highlight the simultaneous trends of refamilialization and defamilialization among the policy actors. Following this role division, the following explores the parties’ actions and interactions throughout the policy formulation, execution, and evaluation stages.

### 4.2 Trends in China’s childcare policies

First, family policy actions highlight the refamilialization trend in China’s childcare policies, where families assume the primary childcare role. This study’s two-dimensional analysis reveals that families are the key agents responsible for child health and hygiene. Policies seek to promote scientific parenting and emphasize children’s physical and mental wellbeing through avenues such as online classes, so as to strengthen family caregiving capabilities. This trend is significantly shaped by traditional Confucian family ethics and the prevailing Chinese view of the family as the principal caregiver. Traditional Chinese family values also stress kinship, respect for the older adult, and care for the young. As such, children depend on parental support during their early years and subsequently provide care for their aging parents. Familial beliefs also influence the formulation of childcare policy objectives. For example, the GOPD asserts that childcare for children aged under 3 years should be family-centered, with the use of supplementary care services. Under this principle, the development of infant and toddler care services should focus on offering scientific parenting guidance to families and essential support for those experiencing caregiving challenges.

Second, China’s socioeconomic development has led to a defamilialization trend in childcare policies. As more women join the workforce, responsibilities for child health, which were traditionally assigned to women, are now being transferred to other family members, and governmental and institutional benefits are redistributed. Defamilialization refers to the degree to which familial welfare responsibilities are transferred to entities outside the family through both public and market pathways (46). Regarding the public pathway, the government plays a central role in childcare policies. With child health as the primary objective, the government’s focus has recently shifted from declining birth rates and disease prevention to the establishment of a comprehensive child health service system. As the authoritative political entity, the government surpasses policy actors’ limitations related to concepts, capabilities, and status. Moreover, the government plays a vital role in organizing, coordinating, and balancing policy actors’ interests to enhance childcare inclusivity that is characterized by mutual benefits and the fostering of social harmony. Regarding the market pathway, childcare institutions are pivotal actors in terms of policy actions related to safety management, health and hygiene, and scientific parenting. The government has established standards for institutions and has implemented registration and review processes. For example, on July 8, 2019, the National Health Commission’s Population and Family Department issued the *Regulations on the Management of Childcare Institutions (Trial) (Draft for Comments)* and *Standards for the Setup of Childcare Institutions (Trial) (Draft for Comments)*. As of February 28, 2024, data from the Population Monitoring and Family Development Department of the National Health Commission indicate that nearly 100,000 institutions currently provide childcare services, with approximately 4.8 million available places.^6^ This demonstrates that China’s childcare institutions are evolving in a way that promotes child health and wellbeing.

Third, the current state of China’s childcare policies reflects the simultaneous presence of refamilialization and defamilialization trends. This is particularly evident based on two key aspects. First, childcare policies tend to promote optional familialism, under which the government and institutions provide childcare services while offering maternity leave and subsidies for parents caring for children at home (47), and parents can choose between public and family care. Second, certain childcare policies incorporate defamilialization strategies while displaying refamilialization characteristics. These initiatives, primarily implemented by social organizations and communities, offer universal community-based childcare, provide care subsidies, and extend parental leave, so as to foster family development (48). Countries such as Germany and Japan, which view the family as the primary childcare provider, actively support family caregiving roles by establishing comprehensive allowances and parental leave systems (49, 50). Conversely, they also promote a systematic approach to social care policies that encourage the government to assume greater childcare responsibility. China is still exploring developmental models of childcare policies. However, the defamilialization and refamilialization models of public family services are not mutually exclusive but coexist and interact with each another (37). Therefore, public policies must balance these two trends with greater precision. The defamilialization trend requires the government to expand public childcare service availability and increase childcare institutions’ financial support. This will ensure the accessibility of affordable and high-quality childcare services across different Chinese regions. During this process, the government must act as both the resource provider and policy implementation regulator to ensure that institutions adhere to health and safety standards and scientifically-based child-rearing practices. Simultaneously, the ongoing refamilialization trend means that policy designs cannot overlook the critical role of families in childcare. Therefore, the government, alongside social organizations and communities, should adopt more flexible approaches toward offering families closer complementary and supportive childcare services in order to address disparities in healthcare quality, standards, and childcare resources. Further, it is essential to pay attention to family perspectives and meet their needs within the policy framework.

Finally, the shortcomings of China’s childcare policies are reflected through community-based services’ restricted capacity, highlighting a crucial area for the future enhancement of policy actors’ roles. The two-dimensional analysis reveals that social organizations and communities primarily contribute to fostering a supportive childcare environment. However, their impact on child safety management, health, and scientific parenting remains limited. The success of childcare policies in promoting child health depends on their ability to systematically identify and address social determinants and ensure that families have access to additional community resources (51). The current delivery of quality childcare has several challenges, including urban–rural disparities, an increase in noncommunicable health issues, and inconsistent service quality. These issues exceed the individual families’ capabilities and cannot be resolved solely through market-driven solutions. As such, community initiatives can play a vital role in supporting underprivileged areas and populations by bridging gaps in the public service infrastructure for childcare in rural and impoverished settings and fostering a societal emphasis on child health. Emphasizing inclusive, community-based childcare services can further rectify the shortcomings of market-oriented institutions through strategies such as increasing the availability of community childcare places, enhancing volunteer service quality, and improving training systems for community childcare providers. Positioning child health as a universal social welfare priority rather than a selective benefit can cultivate a socially-engaged environment that can collectively support the healthy development of childcare services.

### 4.3 Limitations of the study

This study’s policy text sample focused on the prefectural level. Therefore, utilizing more detailed county-level data can better capture the nuanced relationships between these factors. Doing so will be particularly pertinent for the Chinese context, where significant developmental disparities exist between districts and counties. Moreover, as additional county-level policies are enacted, the future research should conduct further analyses of this study’s policy texts to enhance the understanding of the regional variations in childcare policies and child health outcomes in China.

This study conducted its qualitative analysis using NVivo, which may have introduced some subjectivity when defining specific policy objective categories. As policies evolve, the future research should incorporate specific scientific indicators into the child health objective framework to offer more detailed guidelines for child health. These indicators could include nutritional status, immunization rates, and utilization of healthcare and community health services, so as to enhancing the rigor and relevance of the results.

## 5 Conclusion

This study presents three key conclusions from its analysis of China’s childcare policies. First, within the childcare policy framework, the government plays a central role as an organizer, accountable entity, and resource provider, thereby establishing itself as the most significant policy actor. Second, China’s childcare policies are undergoing concurrent refamilialization and defamilialization trends and are shifting from government-led initiatives to coordinated efforts by various policy actors. Third, China’s childcare policies focus on establishing three aspects among childcare institutions: safety management, health and hygiene, and scientific parenting. The construction of community-based services is reflected in the creation of a social childcare environment. Therefore, the future childcare policies should focus on the development of community-based service systems.

This study provides several key insights for childcare policy actors. First, the government should clarify the responsibilities and obligations of each policy actor from the legal and policy standpoints to ensure children’s healthy development. This involves providing communities and institutions with policy support, such as tax exemptions, operating subsidies, and skills training for staff. Doing so can help prevent families from bearing an excessive share of childcare responsibilities. Second, communities should establish inclusive childcare institutions. In economically-disadvantaged rural areas with high rates of child illness, grassroots medical units should be encouraged to enter into service agreements with childcare institutions to deliver health management services for infants and young children. These should include child health check-ups, nutritional guidance, and disease prevention measures. Meanwhile, in urban areas, complementary and supportive childcare services should be provided near residential areas; for example, by integrating childcare facilities into new housing development plans. Third, it is crucial to establish platforms that facilitate interaction and communication between diverse stakeholders. Families, as service providers and recipients, should have their perspectives valued. Therefore, creating a platform for childcare dialog will enable families to regularly communicate with government agencies about community childcare institutions’ service quality, problems within institutions, and their overall satisfaction levels. Finally, to ensure effective oversight, the allocation of institutional subsidies should be tied to factors such as the number of complaints received, parental satisfaction ratings, and overall service quality. Ultimately, through efficient collaboration between various policy actors, China’s childcare policies can more effectively support child health and wellbeing.

## Data Availability

The original contributions presented in the study are included in the article/supplementary material, further inquiries can be directed to the corresponding author.

## Footnotes

^1^Convention on the Rights of the Child. Available at: https://www.unicef.org/child-rights-convention.

^2^Implementing the United Nations Convention on the Rights of the Child. Available at: https://www.qub.ac.uk/Research/case-studies/implementing-un-convention-rights-of-child.html.

^3^The above data are from the *China Children’s Development Plan (2001–2010)*, the *China Children’s Development Plan (2011–2020)*, and the *China Children’s Development Plan (2021–2030)*. Available at: https://www.gov.cn/zhengce/xxgk/.

^4^Guiding Opinions on Promoting the Development of Care Services for Infants and Toddlers Under 3 Years Old. Available at: https://www.gov.cn/zhengce/content/2019-05/09/content_5389983.htm.

^5^*Notice from the People’s Government Office of Changzhi City on Issuing the Implementation Plan for the Inclusive Child Care Service Development Demonstration Project in Changzhi City*. Available at: https://www.changzhi.gov.cn/xxgkml/zfxxgkml/szfgzbm/srmzfbgt/czsrmzf/zbwj/202403/t20240318_2877562.shtml.

^6^*Nationwide, Nearly 100,000 Childcare Institutions*. Website of the Central People’s Government of the People’s Republic of China, 2024-02-28. Available at: https://www.gov.cn/lianbo/bumen/202402/content_6934815.htm.
